# Recent Advances in the Role of Bacteriophages in the Aetiology and Therapy of Vaginal Dysbiosis in the Form of Bacterial Vaginosis and the Prevention of Preterm Birth

**DOI:** 10.3390/microorganisms13102410

**Published:** 2025-10-21

**Authors:** Ronald F. Lamont, Amaan Ali, Jan Stener Jørgensen

**Affiliations:** 1 Research Unit of Gynaecology and Obstetrics, Department of Gynecology and Obstetrics, Institute of Clinical Research, University of Southern Denmark, Kløvervænget 10, 5000 Odense, Denmark; jan.stener@rsyd.dk; 2 Division of Surgery, Hillingdon Hospital NHS Foundation Trust, Pield Heath Road, London UB8 3NN, UK; aliamaan@hotmail.co.uk

**Keywords:** aetiology, birth, bacterial vaginosis, biochemistry, dysbiosis, microbiology, phage virus, prediction, prevention, preterm, probiotics, sexual transmission, treatment, vagina, virome

## Abstract

Bacterial vaginosis is more than a mild inconvenience for women and has been shown to be an important cause of morbidity and mortality in women through sexually transmitted infections, and in babies due to late miscarriage and preterm birth. The aetiology of bacterial vaginosis remains unclear but there is increasing evidence to support sexual transmission as a cause. Preterm birth is a major cause of neonatal and perinatal mortality and morbidity worldwide and a huge cost on healthcare. The earlier bacterial vaginosis is detected in pregnancy, the greater the risk of an adverse outcome like preterm birth. Bacteriophages influence the vaginal microbiome, resulting in a eubiotic or dysbiotic state that may have implications on the prediction and prevention of preterm birth. We have provided the evidence to link vaginal dysbiosis in the form of bacterial vaginosis with the prediction and prevention of preterm birth. We have also explored the role of bacteriophages in bacterial vaginosis and the possibility of therapeutic interventions. Bacteriophages play an important role in the aetiology of vaginal dysbiosis and novel therapeutic interventions may help in the prediction and prevention of preterm birth through achieving vaginal eubiosis.

## 1. Background

### The Importance of Bacteriophages

Bacteriophages are obligate intracellular bacterial parasites that employ the biosynthetic machinery of the host to live and multiply. Bacteriophages are also very host-specific, such that phage typing is used diagnostically to identify isolates of different bacterial species [1] and therapeutically for specific bacterial infections [2]. This is particularly helpful in low- or medium-income countries where the development and introduction of new antibiotics is too costly [3]. Bacteriophage specificity has additional advantages over traditional antimicrobials. By targeting specific pathogenic bacteria, dysbiotic microbiota and secondary bacterial overgrowth and its sequelae can be prevented, e.g., *Clostridium difficile* and pseudomembranous colitis or *Gardnerella vaginalis*, *Lactobacillus iners*, and bacterial vaginosis (BV) [4,5]. Bacteriophage therapy has re-emerged to challenge bacterial resistance [6] and can be used naturally or as synthetic bacteriophage cocktails or in combination with antibiotics. The specificity of bacteriophages permits the sparing of important co-existent non-pathogenic commensals that contribute specifically to long-term vaginal eubiosis. Because of the inherent margin of safety, adverse effects are minimised, and nature’s response is amplified with less frequency of dosage compared to antibiotics [7,8,9]. Bacteriophages may also be used to investigate and treat biofilms [10], which is an important feature of BV [11], with no side effects. The structure and function of bacteriophages, their use in industry, and the life cycle of bacteriophages, as this pertains to the BV syndrome, has been summarised elsewhere [12].

## 2. Vaginal Eubiosis and Dysbiosis

The vaginal microbiome is composed of a core and variable group of organisms [13], which are influenced by transient community members and host factors, such as age, race, lifestyle, physiology, genotype, immune status, environment, and sexual activity [14].

### 2.1. Vaginal Eubiosis

The vaginal mucosa is the first barrier of protection against pathogens. A healthy vaginal microbiome (vaginal eubiosis) is an important component of this defence and provides protection against several urogenital conditions, such as BV, candidiasis, urinary tract infections, and sexually transmitted infections (STIs), including viral infections such as human papilloma virus (HPV), human immunodeficiency virus (HIV), and herpes simplex virus (HSV) [15]. Much of our knowledge about the composition of the vaginal microbiota is derived from semiquantitative and qualitative studies using culture-dependent techniques. Culture and microscopy of the vaginal microbiota from healthy, asymptomatic women is characterised by a predominance of lactic-acid-producing bacteria, mainly, though not exclusively, from the genus *Lactobacillus*. Vaginal lactobacilli promote a healthy vaginal milieu through several properties, such as providing numerical dominance, prevention of adhesion, and production of antimicrobial compounds, such as lactic acid, H_2_O_2_, bacteriocins [16], and probiotics [17]. This helps to maintain the acid environment at a pH of <4.5 that is inhospitable to many bacteria and negatively correlated with BV. This is vaginal eubiosis. Prior to molecular-based techniques, and using only culture and microscopy without fermentation tests, lactobacilli were only identifiable to the genus level. Accordingly, it was not possible to identify a *Lactobacillus* to the species level and hence not possible to assess species-specific antimicrobial properties. As lactobacilli are taxonomically complex and composed of 170+ species that cannot be easily differentiated phenotypically, it is not surprising that investigators thought that no two women had the same complement of lactobacilli in the vagina [18]. With the introduction of the Human Microbiome Project (HMP) [13], new information from molecular-based culture-independent techniques have become available and our knowledge of the vaginal microbiome has been greatly expanded and provided information that has changed the way we define the vaginal microbiota [19]. Using molecular technology, we now know that globally, a woman’s vagina is colonised by one or two species of *Lactobacillus* from a short list of four: *L. crispatus*, *L. gasseri*, *L. iners*, and *L. jensenii*, corresponding to community state types (CSTs) I, II, III, and V. The species that belong to CST IV were dominated by non-*lactobacillus* bacterial spp., such as *Prevotella*, *Dialister*, *Sneathia*, *Megasphaera*, *Eggerthella*, and *Atopobium*, a microbiota that is typically associated with BV [20]. Though the application of phylogenetic taxonomic procedures led to improvements in the classification of bacteria assigned to the phylum *Actinobacteria*, further clarification and revision of this large phylum proved necessary. This led to the reconstruction of phylogenetic trees and proposals for the recognition of two orders, ten families, and seventeen genera, as well as the transfer of over a hundred species to other genera. As a result, *Atopobium vaginae* has now been renamed *Fannyhessea vaginae* [21].

### 2.2. Vaginal Dysbiosis

While CSTs IVa and IVb represent the dysbiotic types of vaginal microbiota, other forms of vaginal dysbiosis are likely to exist. To distinguish between the different forms of vaginal dysbiosis, henceforth in this review, the term vaginal dysbiosis will signify the form of vaginal dysbiosis associated with BV rather than with candidiasis, STIs, or other abnormal microbiota, such as trichomonas or organisms not commonly found in the vagina like *Staphylococcus aureus* [22], *Haemophilus influenzae* [23], or virulent strains of bacteria frequently found in vaginal eubiosis, such as *E. coli* [24] or *G. vaginalis* [25].

## 3. Bacterial Vaginosis

### 3.1. The Importance of BV

**Figure 1:**
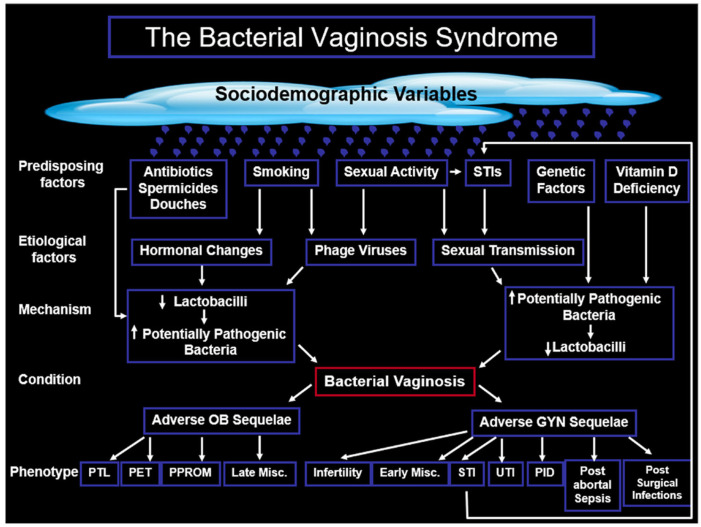
The proposed interaction among the mechanisms, aetiology, predisposing factors, and phenotypic outcomes in obstetrics and gynaecology of the bacterial vaginosis syndrome (with permission from RF Lamont) [12]. GYN, gynaecological; Misc, miscarriage; OB, obstetric; PET, pre-eclampsia/toxaemia; PID, pelvic inflammatory disease; PPROM, preterm prelabour rupture of the membranes; PTL, preterm labour; STI, sexually transmitted infection; UTI, urinary tract infection. ↓: decreased and ↑: increased.

BV is a complex condition now often referred to as “the bacterial vaginosis syndrome”. It is an enigmatic and complex condition where the aetiology remains unexplained; the microbiology differs from case to case, and the antibiotic response with respect to cured/improved, persistence or recurrence is unpredictable, and the phenotypic outcomes differ between individuals. BV is the most common cause of vaginal discharge in high-income countries and the primary cause of vaginal infectious morbidity worldwide. BV is also associated with several sociodemographic variables, such as younger age, black race, and cigarette smoking. Predisposing factors include sexual activity habits and vitamin D deficiency; and aetiological factors include hormonal changes and phage viruses. The mechanisms of BV may be due to a primary decrease in lactobacilli followed by a secondary increase in potentially pathogenic bacteria, or vice versa. BV is also associated with several adverse sequelae in both obstetrics and gynaecology. Obstetrically, these include pre-eclampsia, postpartum endometritis, preterm prelabour rupture of the membranes (PPROMs), late miscarriage (LM), and preterm birth (PTB). In gynaecology, these sequelae include early, late, and recurrent miscarriage, post-abortal sepsis, surgical site infections, post-hysterectomy vaginal cuff infections, urinary tract infections, pelvic inflammatory disease, and infertility [26]. How these factors interact with each other is shown in Figure 1. BV has also been associated with the acquisition of STIs, such as trichomoniasis, gonorrhoea, chlamydia, high-risk HPV [27], and HSV [28]. In addition, BV is also independently associated with seroprevalence of and increased susceptibility to sexual transmission of HIV [29,30,31].

The importance of BV is further manifested in the response to US legislation. In 2013, the CDC issued a report entitled “Antibiotic Resistance Threats in the United States, 2013.” This report introduced concerns in the USA about the growing threat of antibiotic-resistant infections, stating that at least two million people in the US suffer a resistant infection each year, resulting in ~23,000 deaths. The report categorised threats as “urgent,” “serious,” and “concerning,” and highlighted the need for action to prevent infections from becoming untreatable. Concerns were expressed about the lack of development and introduction of new antibiotics. Similar concerns were expressed in the UK [32]. There were an insufficient number of new drugs to replace ineffective antibiotics. The reason given for this was that many large pharmaceutical companies were no longer developing new antibiotics, and preferred to focus on new, more profitable long-term medications. In 2012, as an incentive for drug manufacturers to develop new antibiotics, legislation was already signed into US law as a part of the FDA Safety and Innovation Act called the GAIN Act (**G**enerating **A**ntibiotic **I**ncentives **N**ow). This act extended the exclusivity period to market and sell their drug without competition from other pharmaceuticals by five years. This allowed for antibiotics that were used to treat serious or life-threatening infectious conditions to be sold without generic competition. Drugs that qualified for the provisions of the GAIN Act would have the advantage of an expeditious regulatory approval process through the FDA and receive fast-track and priority-review status (see: The Pew Charitable Trusts. *GAIN: How a New Law is Stimulating the Development of Antibiotics* (2013). Available online: http://www.pewhealth.org/other-resource/gain-how-a-new-law-is-stimulating-the-development-of-antibiotics-85899518481#sthash.mPWCEg9C.dpuf (accessed on 21 April 2014)).

One such drug was secnidazole, which is a nitroimidazole structurally related to metronidazole and tinidazole for the treatment of BV as a single oral dose and with a longer serum half-life than metronidazole. While the initial result of a licencing application failed to permit processing under the GAIN Act, discussions in the US Congress were held, and consideration was given to the association between BV and HIV, and STIs and PTB, and the potential for increased mortality and serious morbidity. As a result, the FDA subsequently concluded that BV was a serious condition and accordingly, drugs developed to treat BV merited consideration through the GAIN Act. Secnidazole was approved by the FDA on 15 September 2017, and is now one of the antibiotics recommended by the CDC for the treatment of BV [33].

### 3.2. The Microbiology of Bacterial Vaginosis

In 1955, Gardner and Dukes from Baylor College, Texas published their seminal paper ‘A Newly Defined Specific Infection Previously Classified “Non-specific” Vaginitis’ [34]. Prior to that time, any vaginal discharge not due to gonorrhoea or chlamydia was labelled non-specific vaginitis (NSV). Herman Gardner, an obstetrician and gynaecologist, in his clinical practice, was seeing many women labelled as having NSV. They complained about the same grey, adherent, offensive vaginal discharge; and on Gram-stain microscopy, Gardner was seeing the same small, pleomorphic, Gram-negative bacilli in most women. However, microbial cultures varied in the isolation of a large, and unrelated group of bacteria that included coliforms, diphtheroids, micrococci, and spp. of *Staphylococci* and *Streptococci*. Gardner and Dukes could not believe that the same condition, with the same consistent signs and symptoms and the same appearance on Gram-stain microscopy could result in such a wide range of different culture results. Accordingly, Dukes, a microbiologist was given the task of trying out various culture media under different atmospheric conditions for different periods of time to identify the responsible organism. After 48 h in 10% sheep agar and at low oxygen tension, numerous minute transparent colonies, that were only visible in reflected light, were detected. Gram-stain microscopy of the colonies showed bacilli like those identified by Gardner on the microscopy of his previous patients. The identification was repeated with 13 of the next 14 specimens that Dukes analysed for Gardner. As a result, Gardner and Dukes declared that this microorganism, which they named *Haemophilus vaginalis*, was the monoetiologic agent for NSV; it was then called *H. vaginalis* vaginitis. Now known as BV, this is considered a polymicrobial condition characterised by a decrease in the amount of protective lactic acid producing spp. of *Lactobacillus* and a 1000-fold increase in bacterial diversity among potentially pathogenic organisms. These facultative and strict anaerobes include spp. of *Mobiluncus*, *Bacteroides*, *Peptostreptococcus*, *Gardnerella*, *Sneathea*, *Prevotella*, *Atopobium vaginae*, (now known as *Fannyhessea vaginae*), and others, referred to as BV-associated bacteria (BVAB) [35].

### 3.3. Communication Between the Gut and the Vaginal Microbiome

Debates have been raised about the communication between the gut and the vaginal microbiome, particularly about how this might apply to the use of oral probiotics and whether these could change the vaginal microbiome. The microbiome of any anatomical site is based upon abundance and diversity. The eubiotic gut microbiome is characterised by a very diverse microbiota. In contrast, the eubiotic vaginal microbiota is characterised by a limited diversity of the microbiota [19]. Because of their proximity, there is good evidence to demonstrate that there is a close association between the rectal and vaginal microbiota. The microbiota of the gut and genital tract in women displays complex biological ecosystems that continuously communicate with each other. The crosstalk between these two ecosystems has an impact on the host’s physiological, immunological, and metabolic homeostasis [36]. This is particularly relevant with respect to BV in black women [37,38]. Furthermore, using 16S rRNA gene sequencing, changes in the vaginal microbiota following antimicrobial and probiotic therapy can be detected and provide a rationale for the use of oral probiotics for the treatment of BV [39].

### 3.4. The Biochemistry of Bacterial Vaginosis

The major organic acid metabolite produced by spp. of *Lactobacillus* (lactic acid) has antimicrobial, antiviral, and immunomodulatory properties and is primarily responsible for the acidification of the vagina. At a pH < 4.5, *Lactobacillu* spp. are particularly efficient at producing H_2_O_2_ which releases hydroxyl radicals and chloradinium ions, both of which are toxic to bacteria. With increasing alkalinity, (menstruation, sexual intercourse, douching), or due to antibiotic therapy or changes in endocrine status, the ability of *Lactobicillus* spp. to produce H_2_O_2_ is diminished. This results in increased numbers of *Mobiluncus* spp. and other anaerobes listed in Section 3.1 above. Through a process of synergism, these organisms produce ketoacids such as succinic acid that blunt the chemotactic response of polymorphonuclear leucocytes (hence vaginosis rather than vaginitis) and decrease their killing ability. This leads to further growth of *Mobiluncus* spp. and other anaerobes and leads to a vicious circle of increased growth of potential pathogens. These potentially pathogenic anaerobes, produce aminopeptidases and decarboxylases that break down peptides to amino acids and then to amines, like putrescine, cadaverine, and trimethylamine. At low vaginal pH, these amines remain stable but at a high vaginal pH as in BV, the amines become volatile and give off an unpleasant odour [19].

The species specificity for H_2_O_2_ production by *Lactobacillus* spp. is inversely proportional to the prevalence of BV. While 95% of *L. crispatus* and 94% of *L. jensenii* strains produce H_2_O_2,_ BV was only present in 9% and 7% of women, respectively. In contrast, only 71% of strains of *L. gasseri* and 9% of strains of *L. iners* produce H_2_O_2_, and BV was present in 43% and 36% of women, respectively [40].

The biochemistry of lactic acid production by probiotic spp. of *Lactobacillus* in vaginal eubiosis compared to dysbiosis has been explored. In vaginal eubiosis, the pH is low (≤4.5) with high levels of lactic acid (~110 mM) compared to dysbiosis (>4.5 and <20 mM), respectively. Short chain fatty acids, such as acetic, succinic, butyric, and propionic acids, were low in vaginal eubiosis (<1–4 mM) compared to dysbiosis (2–120 mM). This led investigators to conclude that “the association of high lactic acid levels with *Lactobacillus*-dominated microbiota suggests that this organic acid metabolite contributes to the beneficial properties ascribed to lactobacilli, such as decreased susceptibility of the human host to urogenital pathogens, which would be a desirable characteristic for a vaginal probiotic” [41].

The study also showed that distinct species of *Lactobacillus* acidify the vagina to different pH levels and that the acidity achieved by *L. crispatus* was greater than *L. gasseri*, *L. jensenii*, and *L. iners*. In addition, the production of the D- and L-isomers of lactic acid and the D:L ratio differed between species of *Lactobacillus*. Both *L. gasseri* and *L. crispatus* produced the L- and D-isomer, whereas *L. jensenii* produced only the D-isomer. *L. iners* only produced the L isomer. This suggests that the D-isomer of lactic acid has a protective role. Furthermore, the lactic acid molecule exists as a protonated (neutrally charged) ion when the H^+^ is non-dissociated or a lactate anion (negatively charged) when the H^+^ is dissociated. The protonated form has immunomodulatory and antimicrobial properties compared to the lactate anion and the protonated form of lactic acid predominates at a pH < 3.9 [41].

In conclusion, the more acid present in the vagina, the more efficient the immunomodulatory and antimicrobial effect of probiotics and their metabolites. *L. crispatus* appears to be the most efficient species of *Lactobacillus* to acidify the vagina through lactic acid. Lactic acid, at the same vaginal pH as that achieved by acetic acid or hydrochloric acid, is associated with a non-inflammatory cytokine profile. These properties vary according to the L- or D-isomer of lactic acid and the lactic acid ion (protonated vs. anion). In contrast, a dysbiotic, polymicrobial vaginal microbiota, such as BV, predisposes to a pro-inflammatory milieu that increases the risk of acquisition of HIV/HSV and other STIs.

### 3.5. Molecular Methods of Diagnosing Bacterial Vaginosis

Historically, the diagnosis of BV relied upon non-molecular tests like the Amsel clinical criteria, Nugent score on Gram-stain microscopy of vaginal smears, or commercial point-of-care tests. However, these tests are not readily available, lack objectivity, may be unpleasant to perform, and do not differentiate between the different CSTs and other subtypes of vaginal dysbiosis. With new information from cultivation-independent, molecular-based techniques, there is increasing interest in the use of molecular technology for the diagnosis of BV. Currently, several commercially available molecular diagnostic tests are available, many of which have CE-IVD (Conformité Européenne in vitro diagnostic) and/or FDA (US Food and Drug Administration) approval [25]. These molecular tests perform well against standard microscopy with respect to sensitivities and specificities. They also have the advantage of quantification, detection of fastidious organisms, objectivity, and validity for self-obtained vaginal swabs. We still do not know whether dysbiosis due to BV is because of the presence of potentially pathogenic bacteria or the absence of eubiotic bacteria, or a combination of both. As users of the technology become better educated in its potential, the importance of including bacteria that are positive predictors as well as those that are negative predictors of BV has influenced the choice of candidate bacteria to be included in the molecular test. Common choices of candidate bacteria that are positive predictors of BV are *F. vaginae* [42], *G. vaginalis*, *Megasphaera* spp. (subtypes 1&2), and BVAB-2 (now known as *Oscillospiraceae bacterium* strain CHIC02 [43]). The bacteria that are commonly used as negative predictors of BV are *L. acidophilus*, *L. crispatus*, *L. jensenii*, and *L. gasseri.*

Using molecular techniques, when detection of *L. crispatus* was combined with *G. vaginalis*, *F. vaginae*, or BVAB-2, their sensitivities and specificities for the diagnosis of BV were 99.3% and 97.8%, 98.9% and 98.7%, 98.2% and 98.5%, respectively [44]. This emphasises that it is difficult to diagnose BV using only one species, yet using combinations of *L. crispatus* with *G. vaginalis* or *F. vaginae* demonstrated a better diagnostic approach, particularly the combination of *L. crispatus* and *G. vaginalis* (see Table 1). The use of quantitative assessment of microbial abundance, the diversity of organisms present, specific primers for different gene sequence regions, and the clades and biovars of target microbes should be recognised and incorporated into future molecular diagnostic tests to differentiate between vaginal eubiosis and dysbiosis.

### 3.6. Sexual Association or Sexual Transmission of BV

For many years, BV has been known to have a sexual association. The younger the age at sexual debut, the greater the number of lifetime sexual partners; and a recent change in sexual partner is associated with an increased risk of developing BV. In addition, in sexually active women, the use of an intrauterine contraceptive device (IUCD) and vaginal douching are also associated with BV. Historically, clinicians were reluctant to label BV as an STI because this might deter women with BV from seeking help for investigation and treatment. One of the reasons given for the assumption that BV was not sexually transmitted was that it was detected in a *Virgo intacta*. Another reason was that in women who have sex with women (WSW), there was a 30% prevalence of BV; and finally, there appeared to be no benefit of treating the male partner. However, these defences and assumptions have been questioned. The veracity of claims of virginity has been doubted. Investigators suggested that male partners of women with BV might have been less than conscientious in taking high doses of metronidazole which caused sickness and necessitated abstinence from alcohol. In addition, only a few such studies recorded observed consumption and/or counted return of unused medications. Finally, Gram-staining of a vaginal smear identified that in faithful couples of WSW, there was a high correlation in the vaginal microbiota of both partners, which is likely to reflect the shared use of sex toys [45].

In their seminal paper in 1955, Gardner and Dukes were in no doubt that their detection of *H. vaginalis* as a cause of vaginitis was a sexually transmitted infection [34]. This has been strengthened by anecdotal evidence from many practitioners experienced in the investigation and treatment of BV. Currently, there is now strong evidence to support treating the sexual partner of women with BV because of sexual transmission of diseases [12,35,45,46,47] and also continuing evidence of the association between BV and STIs [48], particularly HIV acquisition and transmission [49].

## 4. The Role of Bacteriophages in the Aetiology of Bacterial Vaginosis

*Lactobacillus* spp. are widely used in the dairy industry for their putative health value and hence have been considered for their potential in healthy probiotic diets [50]; by 2009, over 230 *Lactobacillus* phage viruses had been identified [51]. The majority of clinical trials addressing the use of vaginal probiotics for the prevention of BV have exhibited clinical efficacy. They have demonstrated comparable efficacy of intermittent metronidazole treatment with a two-month course of probiotics containing several strains of *Lactobacillus* for the prevention of BV [52]. However, one of the most common reasons for the failure of large-scale industrial fermentation is attack from lytic bacteriophages. In a controversial study, a brand of yoghurt containing a strain from the *L. acidophilus* complex colonised the vagina and was successful in treating vaginal yeast infection [53]. Although promising for future use as a probiotic, the strain disappeared and is no longer identifiable. The disappearance of the strain was thought to have been due to natural causes, such as a virulent lytic phage. It was postulated that this was released by a successor strain that eradicated its predecessor. This would support the hypothesis that bacteriophages may parasitise and reduce the quality or quantity of eubiotic species of *Lactobacillus* that may result in recurrent or resistant BV. An elegant study evaluated the stability of dairy *L. acidophilus* cultures, and whether such *Lactobacillus* spp. could be inhibited by bacteriocins or bacteriophages released by other dairy spp. of *Lactobacillus*. The authors concluded that “such *Lactobacillus* probiotic products may be unstable or unsafe because they could be inhibited by phages or bacteriocins or because they release them to inhibit the *lactobacilli* of other dairy products” [54]. Phage-mediated lysis of vaginal lactobacilli might result in a significant decrease in the quantity or quality of lactobacilli and the subsequent growth of high numbers of anaerobic bacteria. In one in vitro study, the rate of bacteriophage detection was less in healthy women and higher in those women with BV or vulvovaginal candidiasis. Accordingly, it was postulated that the bacteriophages detected might infect vaginal lactobacilli from the same or different women and this has implications on the possible role of sexual transmission of bacteriophages. In addition, bacteriophages isolated from a human intestinal strain of *Lactobacillus* were able to lyse vaginal lactobacilli, which suggests that some of the vaginal bacteriophages may be derived from the lower gastrointestinal tract [55].

In a vaginal microbiome analysis of women undergoing in vitro fertilisation for male factor infertility, a comparison between BV-positive and BV-negative women revealed statistically significant viral β-diversity. This indicated a different viral community in BV-positive and BV-negative samples [56]. An identifiable co-abundance pattern was seen between bacterial and phage populations. This permitted analysis of the virome and prediction of the bacterial CST together with the presence of BVAB that were either pathogens or protective against BV. BV-negative samples had a greater abundance of bacteriophages that targeted species of *Lactobacillus* compared to BV-positive samples. A high abundance of anaerobic bacteria was negatively associated with the presence of *Lactobacillus*-infecting bacteriophages. This suggested that a low abundance of *Lactobacillus* resulted in fewer specific *Lactobacillus* bacteriophages and vice versa with a low abundance of pathogenic BVAB and their associated bacteriophages.

The results suggested that it was not prophage induction that was responsible for *Lactobacillus*-depleted populations but rather the introduction of novel, external, lytic bacteriophages that may have been acquired from a sexual partner. Alternatively, this might have been due to the absence of protective bacteriophages in the vaginal mucosa which facilitated colonisation by pathogenic BVAB and subsequent vaginal dysbiosis. The presence of bacteriophages specific to *Lactobacillus* in BV-negative samples could suggest the protective role of these bacteriophages in developing *Lactobacillus* strains that are more resilient to external lytic bacteriophages [56].

In a meta-transcriptomic study comparing thirty-one women with BV who responded to metronidazole with six non-responders with persistent BV, post-metronidazole therapy identified upregulated anti-phage genes in isolates of *G. vaginalis* [12]. This indicated a possible vaginal bacteriophage attack [57]. The presence of lytic bacteriophages in the vagina may reduce the effectiveness of metronidazole which conserves lactobacilli that may be a host for these bacteriophages, and result in recurrence. This observation was confirmed in a study in which microscopy and rRNA-sequencing analysis of the vaginal microbiome was conducted before and after treatment with oral metronidazole (500 mg, b.d) in Rwandan women with BV. A surprisingly low cure rate of only 54% was observed with metronidazole and in many cases, a preserved and increased population of *Lactobacillus* spp. [58].

This series of studies demonstrates that bacteriophages released from the vaginal lactobacilli of some women can infect the lactobacilli of other women under in vitro conditions [12]. Since intestinal strains of *Lactobacillus* lysed multiple isolates of vaginal *Lactobacillus* spp. [55], it may be that the lower intestine is a reservoir for bacteriophages that infect vaginal lactobacilli by the recto-vaginal route. Alternatively, the route may be through sexual transmission. In a 1991 editorial, Dr Anona Blackwell found the concept of bacteriophages causing BV interesting, but not consistent with the possibility that the condition was sexually transmitted [59]. This highlights the possibility that vaginal lactobacilli have bacteriophages [60] and that *Lactobacillus*-derived bacteriophages from dairy products can lyse vaginal spp. of *Lactobacillus* [55] (see Section 6.1 below). However, she considered that it was possible that temperate, diet-acquired bacteriophages induce bacteriophages to become lytic by some other factor related to sexual activity. Alternatively, it may be that *Lactobacillus* bacteriophages are directly inoculated into the vagina from male or female partners [59,61]. Smoking is a risk factor for BV, and chemicals in cigarette smoke can be found in vaginal secretions that induce lytic conversion of *Lactobacillus* bacteriophages [62]. This may help to explain the association between cigarette smoking and BV [63].

### 4.1. Potential Beneficial Effects of Bacteriophages

The human gut microbiome has been shown to play a crucial role in maintaining the delicate balance of human health. As in the vagina, disruption of the microbiome can result inter alia in the development of inflammation and other disorders. The gut phageome is still considered the “dark matter” of the gut, since more than 80% of viral genomes remain uncharacterised and it is likely that the same applies to the vagina [64]. In contrast to phage-mediated lysis of lactobacilli that may lead to BV, bacteriophages might also be therapeutic, sparing and protecting beneficial species of *Lactobacillus* and promoting vaginal eubiosis [12]. There is no specific category of bacteriophages that are inherently protective of lactobacilli. However, some phage-resistant spp. of *Lactobacillus* are protective, and by selecting such bacteria that have developed resistance to damaging bacteriophages, the probiotic function of these *Lactobacillus* spp. can be preserved. This is evident in the food industry, in which virulent *Lactobacillus* bacteriophages can cause costly spoilage. To control this natural phenomenon, the industry has tried several measures; yet, despite these efforts, bacteriophages continue to evolve, and new variants emerge. To keep up with this evolution of bacteriophages, continued research is necessary to better understand bacteriophage diversity and host interaction. This might improve the selection of lactic-acid-producing strains of *Lactobacillus* and optimise anti-phage mechanisms [65].

### 4.2. Bacteriophage Protection of Lactobacillus spp.

Phage viruses provide protection for *Lactobacillus* spp. through several mechanisms. Firstly, they may provide immunity against superinfection by integrating their genome into the host bacterium and entering a dormant, lysogenic, temperate state. Subsequently, this integrated prophage can express genes that protect the bacterium from infection by closely related bacteriophages. In the future, bacteriophages may be used as specific modulators of the human microbiome in a variety of infectious and non-communicable human diseases [66]. Bacteriophages also target and destroy pathogens like *G. vaginalis* which indirectly protect the beneficial spp. of *Lactobacillus* by creating a less competitive environment and preserving an environment favoured by lactobacilli. Bacteriophages may also drive bacterial evolution [67]. As a result of constant attacks from bacteriophages, bacteria evolve defence systems such as the CRISP-Cas system [68]. Such adaptive immune systems provide “memory” and subsequent defence against specific bacteriophages. In this way, resistant strains of *Lactobacillus* are better equipped to withstand bacteriophage attack. Finally, “natural selection” may contribute to the development of robust bacteriophage-resistant strains of *Lactobacillus*. As in higher-order organisms, Darwinian theory dictates that the survival of the fittest is the law of nature and just as the most vulnerable of the herd becomes the primary target of the predator, bacteriophages often specifically target spp. of *Lactobacillus* by culling the most susceptible of strains, which creates a selective pressure for only the strongest to survive. The context in which bacteriophages and bacteria relate depends on the specificity of the bacteriophage and the host bacterium, as well as the environmental conditions. Lytic bacteriophages rapidly replicate and destroy their hosts, whereas temperate bacteriophages are dormant, integrate their host’s genome, and confer protection. It should not be forgotten that while some bacteriophages are protective, specific environmental stressors such as chemicals or cigarette smoking may cause dormant, temperate bacteriophages in lactobacilli to become lytic and adversely affect the population of *Lactobacillus* spp. and hence dysbiosis. While the food industry pursues the commercial consequences of bacteriophages, the protective role of bacteriophages merits further consideration for therapeutics in the healthcare system. Similarly, the combination of bacteriophages with probiotics is an emerging strategy to reduce bacterial infections and improve gut health. Finally, new bacteriophages are being engineered and developed to target harmful bacteria while sparing bacteria like lactic-acid-producing *Lactobacillus* spp. that in certain niches provide beneficial effects [69].

## 5. Cost of Preterm Birth

### 5.1. The Importance of Preterm Birth

The human impact of PTB was first brought to public attention about 25 years ago. Up to that time, spontaneous preterm labour (SPTL) leading to PTB was somewhat of a Cinderella subject because of doubts about aetiology, prediction, prevention, diagnosis, interventions, mode of delivery, and in utero transfer [70]. In 2009, the March of Dimes (MoD) Foundation, based on data from the World Health Organisation, produced a White Paper on PTB [71], providing hitherto unavailable data about the extent of PTB worldwide with respect to the numbers and percentages per population of PTB regionally and globally (Table 2). Somewhat surprising to other high-income countries, North America (mainly the USA) had a high rate of PTB, almost matching that of Africa. These data would influence the US Congress to change their mind years later and grant an antibiotic for the treatment of BV access to the GAIN Act to accelerate registration, etc., (see Section 3.1). The key messages from the MoD were that (i) 28% of four million neonatal deaths annually are due to PTB; (ii) 12.9 million PTBs occur annually worldwide (9.6% of births); (iii) 85% of PTBs occur in Africa/Asia; (iv) the rate of PTB is increasing (by 36% in USA in the previous 25 years and mostly due to late PTB [34–36 completed weeks of gestation]); (v) strategies to reduce death and disability should be given priority under the UN Millennium Development Goals (MDGs), specifically MDG 4 (child survival) and MGD 5 (improvement in women’s health).

### 5.2. The Human Impact of PTB at the Extremes of Viability

In 2001, the EPICure study reported on all births between March and December 1995 at less than 26 weeks of gestation from all maternity centres throughout the United Kingdom and Ireland. Data were gathered on 843 infants who were admitted for neonatal intensive care of whom only 321 survived to be discharged home. It was estimated that of babies born around the limits of viability (22–26 completed weeks of gestation), around 65% died on the delivery suite or the neonatal intensive care unit. Of those who survived to 30 months, 50% were disabled, and in 50% of these, the handicap was severe, such that by that age, only ~12% were alive and intact [72]. In 2009, The EXPRESS Study reported on the one-year survival of extremely preterm infants after active perinatal care in Sweden. This was a population-based prospective observational study between 2004 and 2007, addressing the 1-year survival/morbidity of babies born between 22 and 26 completed weeks of gestation. The study comprised 305,318 infants of which 1011 births occurred at 22–26 weeks of gestation and 707 were liveborn. The mortality by gestational age for liveborn infants is shown in Table 3a,b. Babies born at 22, 24 or 26 completed weeks of gestation had an infant mortality rate of 54%, 21%, and 2%, respectively, and a survival rate without major morbidity at 1-year of 0.02%, 14.1%, and 45.9%, respectively [73].

### 5.3. The Financial Cost of PTB

The data on the financial cost of PTB is limited and difficult to obtain. However, the cost of hospital readmissions in the UK in the first five to ten years of life is 20× greater for those babies born before 28 weeks’ gestation compared to those born > 37 weeks [74]. In 2007, the Institute of Medicine (IOM) in the USA calculated that the annual cost associated with PTB was $26.2 billion. This comprised (i) costs of neonatal care ($16.9 billion); (ii) maternal delivery costs ($1.9 billion); (iii) programmes for children up to age three with disabilities and developmental delays ($611 million); (iv) special education services ($1.1 billion); and (v) loss of pay for parents whose babies had been born preterm ($5.7 billion) [75].

### 5.4. The Psychosocial Cost of PTB

The human and financial costs of PTB above can be measured but they cast a long shadow and the psychosocial costs of PTB remain incalculable.

### 5.5. The Aetiology of Preterm Birth

Historically, PTB was thought to be idiopathic because ~50% of women had no discernible risk factor. However, following detailed analysis of the fetus, mother, and placenta, it was found that in 96% of women, one factor could be identified and in 58%, there were two or more factors present with infection, which is the most common factor (38%) after faulty placentation (Table 4) [76]. The earlier in pregnancy that PTB occurs, the more likely this is to be due to infection [77], and this is the stage of pregnancy at which mortality and morbidity is at its greatest (see Section 4.2).

## 6. The Prediction and Prevention of Preterm Birth

### 6.1. Bacterial Vaginosis in the Prediction of Preterm Birth

Women with BV before 16 weeks of gestation have a higher risk of late miscarriage (LM; birth < 24 weeks gestation) and early PTB (<34 weeks’ gestation) (16.7%) than women who are BV-negative (3.4%) (Odds ratio = 5.35 [95% CI = 2.73–10.5]; Relative risk = 3.12 [95%CI = 2.23–4.37]; *p*-value = 0.000001) [78]. Only 2% of women who do not have BV in the second trimester will develop BV by 34 weeks of gestation. In contrast, 50% of women who have BV in the second trimester will still be BV positive at 34 weeks of gestation [79]. The earlier in pregnancy at which an abnormal vaginal microbiota is detected, the greater the risk of an adverse outcome like LM and PTB. A positive screening test for BV at 26–32 weeks of gestation is associated with a two-fold increased risk of PTB. In contrast, a positive-BV screen in the second trimester carries a near seven-fold increased risk of LM or PTB. In summary, the earlier in pregnancy that vaginal dysbiosis is detected, the greater is the subsequent risk of LM or PTB [80]. In a study to illustrate the frequency of occurrence of LM or PTB, according to the grade of vaginal flora on the Gram-stain of vaginal secretions, the conclusions were that an abnormal vaginal microbiota in early pregnancy, even if this resolves spontaneously or post treatment, causes damage which occurs early and persists [81].

### 6.2. Treatment of BV in the Prevention of PTB

The use of antibiotics for the treatment of BV and prevention of infection-related PTB, the controversies surrounding the heterogeneity of the antibiotic studies, the flaws of the subsequent systematic reviews, the putative errors of further studies that used dubious methodology to produce counterintuitive results that continue to lend support to the myth that no antibiotic used at any time in pregnancy can prevent PTB have been extensively addressed [80] and will be the subjects of a subsequent report (Lamont RF, Joergensen JS. Historical Perspectives and Recent Advances in the Influence of the Vaginal Microbiome on the Prediction and Prevention of Preterm Birth. BJOG [In Press]). In the view of the co-authors, (i) the *appropriate* antibiotics (in our view, clindamycin) (ii) given to the *appropriate* women (in our view, those with objective evidence of vaginal dysbiosis due to BV) (iii) at the *appropriate* time during pregnancy to prevent infection and inflammatory tissue damage (in our view, at <22 weeks’ gestation) significantly reduces the risk of LM (80%) and PTB (40%) [82]. Secondary outcomes from the same systematic review demonstrated that, of those babies born preterm, low birth weight (<2500 g) occurred in 20% of those given antibiotics compared to 80% of those who received no treatment or placebo (*p* < 0.009). Furthermore, there was a 32.5-day difference in mean gestational age at birth in those given antibiotics (*p* < 0.024) compared to those who received no treatment. This delay in delivery is significant because each day gained between 23- and 26 weeks of gestation, is associated with a 3% increase in survival [83]. Of those women given antibiotics, there was a statistically significant reduction (86%) in those who delivered before 33 completed weeks of gestation. In addition, in women with the worst degree of BV (Nugent Score = 10), the incidence of PTB and LM was 5.4% in those who were given antibiotics compared with 35.7% for those who received no treatment/placebo. There was also a ~3-fold increase in the rate of LM or PTB in women with persistent BV compared to women cured of BV (28% vs. 10%) (OR = 2.9; 95% CI = 1.3–5.2). Furthermore, there was a ~10-fold increase in the rate of LM or PTB in women with cured but recurrent BV compared to women with cured BV and no recurrence (15% vs. 2%) (OR = 9.3; 95% CI = 1.6–5.35) [84].

In the words of Aldous Huxley (1894–1963; Proper Studies [1927]), “Facts do not cease to exist because they are ignored”.

## 7. Bacteriophages in the Treatment of Bacterial Vaginosis and the Prevention of Preterm Birth

### 7.1. The Vaginal Virome

While the vaginal bacterial microbiome (bacteriome) is the best-studied component of the vaginal microbiome for some years [19]; until recently, application to molecular-based cultivation-independent methods concerning the vaginal viral microbiome (virome) was limited [56,85,86,87,88]. However, using next-generation sequencing, there has currently been a significant increase in the number of manuscripts that address the vaginal virome and the impact it has on women’s health [89,90,91,92,93,94,95,96,97,98]. For further information about the vaginal virome, the reader is directed to a recent review [99].

The vaginal virome comprises viruses that infect human cells (eukaryotic viruses) and those that infect bacteria (bacteriophages). The majority of vaginal virome studies focus on eukaryotic DNA viruses, which usually comprise <5% of viral sequences found in the human vagina [100] and bacteriophages are the most abundant vaginal viral group. Indirectly, bacteriophages can stimulate the host immune response by the release of bacterial products such as endotoxins during bacterial lysis [100]. Approximately, 20% of bacteriophage genes produce peptidoglycan lyase/hydrolase-related genes, which may facilitate bacterial cell wall degradation and destruction during phage infection [97].

Bacteriophages demonstrate either lysogenic or lytic life cycles. Lytic bacteriophages invade and hijack the bacterial replication machinery to produce thousands of viral progeny which result in lysis and death of the host cell. In contrast, lysogenic bacteriophages integrate the host genome, lie dormant, and replicate when the host replicates. Lysogenic bacteriophages also produce virulence factors that may provide survival advantages to the bacterial host. This is achieved by conferring tolerance to ecological stressors, immunity to infection, increased pathogenicity, and antibiotic resistance [101,102]. However, in an unfavourable environment, lysogenic phages can change their life cycle from lysogenic to lytic, subsume bacterial functions, and cause death of the host bacterium [103]. This change in life cycle can be triggered by bacteriophage “quorum sensing” mechanisms that permit bacteria to coordinate their behaviour based on population density [103,104]. There is an increased prevalence of lysogenic bacteriophages in women with BV. This may provide survival value to their hosts to improve persistence [89]. Lysogeny is also common in *L. crispatus* in up to 77% of clinical isolates [105]. In addition, the range of clinical *Lactobacillus* phages is broad and includes several species of *Lactobacillus*. Accordingly, since phages that are lysogenic in one species may be lytic in another, this may be the mechanism by which *lactobacilli* maintain the stability of the vaginal microbiome [60].

### 7.2. The Vaginal Virome in Health and Disease

The vaginal virome is associated with several adverse events in both obstetrics and gynaecology. These include BV and PTB that have already been detailed in the sections above. Historically, an increase in the diversity of the vaginal bacteriome was found to be correlated with PTB [80,100,106] and animal and human studies have demonstrated that an increase in the vaginal eukaryotic DNA virome also increases the risk of PTB [107,108,109]. However, the vaginal phageome was not assessed and this should be addressed in future studies. Communities within the vaginal phageome differ according to whether women have BV or not [56,89], with either decreased *Lactobacillus* phages [56] or higher levels of phages from *Bacillus* spp. and *Escherichia* spp. [89]. There is recent evidence to support a well-documented role that the vaginal virome plays in vaginal health and disease with respect to infertility and the risk of cervical cancer. These are covered in depth elsewhere and not directly associated with the title of this review, but in summary may contribute to the conclusions of the review.

#### 7.2.1. The Indirect Role of Phages in Preterm Birth

Bacteriophages contribute indirectly to PTB and other adverse outcomes in obstetrics and gynaecology through several pathways, such as altering the maternofetal microbiomes, affecting bacterial virulence, modulating the immune system, and driving bacterial evolution [110,111,112]. Bacteriophages can modulate innate immunity through phagocytosis and/or cytokine response. Using a computer model to predict complex and dynamic interactions, the phageome may play important roles in shaping mammalian–bacterial interactions [113].

Unlike eukaryotic viruses, bacteriophages do not infect human cells, but through their bacteriophage properties, they have an indirect impact on the human host. This being the case, they have a significant effect on pregnancy outcomes. While vaginal eubiosis is typically achieved by lactic-acid-producing *Lactobacillus* spp. that maintain a low vaginal pH, bacteriophages can modulate the vaginal microbiome by infecting and destroying these beneficial *Lactobacillus* spp. This shifts the microbial balance towards a higher diversity of anaerobic bacteria, which is BV. A loss of *Lactobacillus* dominance and an increase in potentially pathogenic or opportunistic anaerobic bacteria is strongly associated with a higher risk of PTB. The presence of these harmful bacteria and vaginal dysbiosis provides a mechanism by producing pro-inflammatory cytokines and chemokines in vaginal fluid, which cause inflammation and contribute to PTB [114] and other adverse outcomes in obstetrics and gynaecology (see Figure 1). Finally, since temperate or lysogenic bacteriophages integrate their genome into a bacterium without killing it, they can sometimes transfer genes to the bacterium, which increase their virulence and their ability to cause disease [115]. This horizontal gene transfer by bacteriophages can donate genes for antibiotic resistance and increase virulence between bacteria. This can lead to more aggressive bacterial strains, which may be more difficult to eradicate and more likely to cause an inflammatory response that can lead to PTB.

#### 7.2.2. Phage Viruses in Infertility

Few studies have examined the role of the vaginal virome in infertility. In women undergoing in vitro fertilisation (IVF), there was a tendency towards a higher diversity in a eukaryotic DNA virome in women whose IVF procedure failed to achieve a clinical pregnancy [116]. In women with infertility, irrespective of whether this was due to male or female factor, a higher prevalence of a specific phage virus (anellovirus) was found [94]. These are small DNA viruses that were thought to be commensals but are often used as markers of immunosuppression. The authors of this study found that in infertility, individuals colonised by this *L. crispatus* phage virus were less likely to have a *L. crispatus*-dominated bacteriome.

#### 7.2.3. Viruses in Cervical Cancer

The most common sexually transmitted virus is HPV. It is also the most prevalent vaginal eukaryotic virus worldwide, and the causative agent of cervical cancer [27]. HPV infects squamous epithelial cells and often spontaneously resolves within 6–18 months. However, in those cases where the infection persists, certain high-risk HPV genotypes carry a high risk of progression to oropharyngeal and anogenital cancers [117]. High diversity bacteriomes and co-infection with other STIs, such as HIV, HSV, and Epstein–Barr virus (EBV), are associated with decreased clearance of vaginal HPV and persistent infection with high-risk oncogenic HPV. Compared to HPV infection alone, co-infection with HPV and HSV also increases the risk of cervical squamous cell carcinoma or adenocarcinoma by nearly 10-fold [118]. HPV release of oncoproteins downregulates several antimicrobial host defence peptides normally secreted in response to bacterial lipopolysaccharides [119]. Accordingly, HPV colonisation may permit overgrowth of more pathogenic bacteria due to the downregulation of host defence mechanisms. Some species of *Lactobacillus* are immune to the antibacterial effects of these peptides and cleave them to provide an amino acid nutrient source for growth [119]. In vitro, *L. crispatus* and *L. gasseri* downregulate the expression of HPV oncoproteins [120], and may play a direct role in preventing HPV virulence.

## 8. Therapeutic Interventions to Induce Vaginal Eubiosis

### 8.1. Probiotics

The influence of probiotics on the vaginal microbiome in the prediction and prevention of PTB has been covered elsewhere (Lamont RF, Joergensen JS. Historical Perspectives and Recent Advances in the Influence of the Vaginal Microbiome on the Prediction and Prevention of Preterm Birth. BJOG [In Press]). Those species of *Lactobacillus* that are not adapted to grow and survive in the vagina, such as *L. plantarum*, *L. brevis* and *L. fermentum*, should be excluded as a suitable candidate probiotic. It follows that those species of *Lactobacillus* that are adapted to grow and thrive in the vagina, such as *L. crispatus*, *L. jensenii*, and *L. gasseri*, should be chosen and suggests that *L. crispatus* is an excellent prospect for the development of a vaginal probiotic.

There is a rationale behind the consideration of using probiotics: (i) the aetiology of BV remains unknown though there is increasing evidence to implicate sexual transmission [35,45,46,47], (ii) the microbiology differs between women using both cultivation-dependent and molecular methods, (iii) therapeutic options are limited because the response to antibiotics is inconsistent and recurrence and reinfection are common, (iv) while the clinical cure rate is 80–90%, recurrence at 6–12 months is ~50%, (v) new drugs are not forthcoming, (vi) phenotypic outcomes differ between women, (vii) a better understanding of “the BV syndrome” as a major form of vaginal dysbiosis is necessary. In a recent systematic review of the efficacy and safety of different drugs for the treatment of BV, both vaginally applied sucrose and probiotics, resulted in a better clinical response than metronidazole [121]. Used in combination with other novel agents, such compounds may help to prevent recurrence.

A phase II study of the treatment of women with ongoing urogenital problems using the *L. crispatus* probiotic (CTV-05) found that out of 40 participants who lacked *L. crispatus* colonisation at enrollment, 36 (90%) were successfully colonised by *L. crispatus* at the follow-up [122]. In a later randomised controlled trial, *L. crispatus* (CTV-05) was applied for two weeks after an initial course of metronidazole gel. Vaginal colonisation with *L. crispatus* occurred in 61% of cases at day 10 but decreased after applications were stopped [123].

Two recent systematic reviews have provided supporting evidence. In the first of these, women given antibiotics followed by intravaginal probiotics were compared with those treated with antibiotics alone. The results showed a lower recurrence rate of BV at three months, and prolonged cure rates at 24 weeks [124]. The other systematic review found that women treated with probiotics experienced a significant increase in BV cure rates and a significant reduction in recurrence rates of BV [125].

Probiotics have also been used to facilitate eradication of female genital tract high-risk HPV (HR-HPV) infection and progression to dysplasia/neoplasia. A hundred women with HR-HPV infection were randomised to receive either intravaginal *L. crispatus* probiotic strain (chen-01) for five months or placebo [126]. At the 6-month follow-up, women who received the intravaginal probiotic had significantly lower HPV viral loads. In addition, these women had decreased cytological abnormalities together with higher carriage of *L. crispatus* and lower bacteriome diversity. This was similar than in uninfected HPV subjects [126]. These examples illustrate the need for further well-designed large-scale clinical trials to validate the impact of adjunctive intravaginal therapies to restore vaginal health.

### 8.2. Antibiofilm Agents

More than 50% of women will experience recurrent BV within six months of treatment and by one year, this figure is ~80% [127,128]. This is partly due to biofilm formation by BVABs, particularly the species of *Gardnerella* and *Prevotella* [129]. The microenvironment of a biofilm is created by biofilm-forming organisms that make it difficult for antibiotics and host antimicrobial compounds to penetrate. A good example of a biofilm is plaque on teeth and on prosthetic joints. For a comprehensive review of the pathogenesis of biofilm vaginosis, the reader is referred to Swidsinski et al. [11]. The inability of antimicrobials to penetrate the biofilm renders it difficult to eradicate bacteria. In vitro and in vivo studies have tested single-agent compounds to reduce or eradicate biofilms, but antibiotics alone are not sufficient. Some antiseptics in vitro, such as iron sulphide and in vivo octenidine (a cationic surfactant), have shown promise, but resistance with repeated exposure remains a problem.

The use of cationic peptides, that are short chains of amino acids that carry a positive electrical charge due to the presence of positively charged amino acids like lysine and arginine, have shown promise in vitro. Their positive charge allows them to disrupt the negatively charged membranes of bacteria, fungi, and some viruses and their antimicrobial properties play a role in the innate immune system of many organisms. Similarly, the synthetic phage endolysin enzyme PM-477 which is engineered to target and eliminate *Gardnerella* spp., without harming eubiotic vaginal bacteria, has shown promise. In addition, plant extracts, such as Thymbra capitata essential oil, (also known as Spanish oregano), demonstrates antibacterial and anti-inflammatory properties and has shown promise in biofilm reduction including polymicrobial biofilms and ex vivo biofilms [129,130]. The pursuit of future treatment continues [131].

### 8.3. Oleic Acid Treatment

The use of unsaturated long-chain fatty acids such as oleic acid have demonstrated antimicrobial activity against Gram-positive organisms and can modulate the composition of vaginal lactobacilli. Similarly, long-chain fatty acids selectively inhibit *L. iners*, including strains resistant to metronidazole; at the same time, they promote the growth of *L. crispatus*. The mechanism functions through the upregulation of gene products in *L. crispatus*, *L. jensenii*, and *L. gasseri*, that do not exist in *L. iners* and BVABs. The therapeutic potential of oleic acid in the treatment, prevention, and recurrence of BV requires further investigation [132].

### 8.4. Phage Lysins

Phage lysins are lytic bacteriophage enzymes. Their function is to cleave bacterial cell wall peptidoglycan to stimulate phage progeny release. Their effectiveness lies in their ability to control bacterial populations on mucosal surfaces by lysis and destroy Gram-positive bacteria. They act synergistically with antibiotics and control pathogenic bacteria in several conditions [133]. Pathogenic strains of Group B streptococcus (GBS) carry prophages whose genes enhance bacterial growth and biofilm formation [134]. This may help GBS to adapt to the vaginal environment and increase pathogenicity in newborns. Recently, EN534-C, which is a new therapeutic recombinant endolysin derived from a GBS prophage, has been shown in vitro to lyse clinical isolates of GBS while sparing eubiotic vaginal lactobacilli [135].

### 8.5. Vaginal Microbiota Transplantation

Another proposed therapeutic intervention for recurrent BV is vaginal microbiota transplantation (VMT). This involves vaginal transplantation of donor vaginal secretions that are of low diversity, including a significant abundance of species of *Lactobacillus*, such as *L. crispatus*, *L. jensenii*, or *L. gasseri* [20], transvaginally into recipients with BV or other vaginal dysbiosis [136]. In one study, four out of five VMT recipients successfully achieved remission of their BV at follow-up with no adverse effects. While VMT is a promising prospect for the treatment and prevention of recurrence of BV, it is not without its drawbacks. Firstly, donors should be thoroughly screened for STIs and other dysbioses to prevent the transfer of pathogens, resistant fungi, or other undesirable vaginal dysbiotic microbiota [137,138]. Bacteriophages may also be transplanted using donor VMT and these have also been used inter alia in faecal microbiota transfer (FMT) to treat inflammatory bowel disease [139]. This could result in the transfer of undesirable genes that promote antimicrobial resistance (AMR) [140] with potentially serious consequences [141]. This is less likely with VMT compared to FMT because of the reduced mucosal surface area and rigid screening protocols. In ~50% of cases, *Lactobacillaceae* genomes from commercial strains contain an integrated prophage with at least four potential AMR genes encoded within the phage genomes [142]. This supports the recommendation to include AMR screening in VMT [136]. Cost and the need for rigorous donor screening to prevent adverse effects may be a challenge for VMT as a potential intervention, and larger clinical trials will be required.

## 9. Conclusions and Future Research

The detection of BV in early pregnancy is significantly associated with LM and early PTB at which stage perinatal and neonatal mortality and morbidity is at its greatest. For women at risk of infection-related PTB, giving them the appropriate antibiotics before 22 completed weeks of gestation significantly reduces the risks of LM and PTB. There remains a debate about whether metronidazole or clindamycin should be the antibiotic of choice, albeit that both are recommended for the treatment of BV [33]. Those who support the use of metronidazole often do so because metronidazole conserves lactobacilli which, due to their protective properties listed above, are perceived as an advantage. In contrast, clindamycin does not spare lactobacilli, and this may be perceived as disadvantageous. However, if the lactobacilli were parasitized by bacteriophages, clindamycin may eradicate the source of recurrent and/or resistant BV. While the vagina may recolonise naturally, it makes the case for combining antibiotic treatment like clindamycin with a probiotic-containing eubiotic species of *Lactobacillus*, that is adapted to grow and thrive in the vagina.

There remains a lack of clarity over the microbiology of BV. Does BV occur due to the presence potentially pathogenic BVABs or the absence of eubiotic species of *Lactobacillus* or a combination of the two? If so, which comes first and what are the stimuli that favour one mechanism over the other (see Figure 1)? Similarly, bacteriophages can be lysogenic in some bacteria and lytic in others and may affect bacteria that induce eubiosis or those that induce dysbiosis. Molecular tests for the diagnosis of BV have identified the need to include bacterial candidate organisms from both BVABs such as *Sneathia*, *Fannyhessea*, *Gardnerella*, and eubiotic species of *Lactobacillus* such as *L. crispatus* [25]. Ravel’s community state types (CSTs) I, II, III, V correspond, respectively, to *L. crispatus*, *L. gasseri*, *L. iners*, and *L. jensenii*. Ravel also listed two other CSTs, namely IVa and IVb. CST IVa contains a non-*Lactobacillus*-dominated microbiota whereas CST IVb is characterised by bacteria that are typically associated with BV such as *Gardnerella* spp. and *Sneathia sanguinegens*. New information from molecular-based, cultivation-independent techniques [19] has demonstrated that BV may have several subtypes over and above CST-IVa and IVb, cited by Ravel et al. [20], and this might help explain the difference in aetiology, microbiology, and response to antibiotics and phenotypic outcomes among women with BV. In this age of metagenomics, the HMP started with the bacteriome of the oral cavity, nasal cavity, gastriointerinal tract, genito-urinary tract, and skin. The intention was to study the physiology and pathology of the microbiome in health and disease, and to find ways of manipulating the human microbiome to influence physiology and introduce therapy [13]. Only recently have we seen publication of reviews relating to the vaginal virome, which may go on to study the vaginal phageome in health and disease, [99] and possibly also the virulome of different organisms that may change the virulence of an organism by upregulating genes that code for certain virulence factors.

Furthermore, we have seen how viruses either directly or through bacteriophages contribute to morbidity in women. We have also addressed the contribution of vaginal viruses to infertility, cervical dysplasia/cancer, and STIs, such as HIV, HSV, HPV, and EBV. However, steps can be taken to reduce the risks of infectious morbidity such as antibiofilm agents, phage lysins, and the use of long-chain fatty acids, such as oleic acid, and these should be studied further in future research. Finally, bearing in mind current challenges and future landscapes for FMT [139], the role of VMT appears to show promise. In addition, like many scientific discoveries, VMT has brought us full circle to complete the cycle initiated by Gardner and Dukes in 1955 in their seminal paper on *H. vaginalis* vaginitis, now known as BV. To satisfy Koch’s postulates, they carried out a form of VMT. Of 13 volunteers who were inoculated with pure cultures of *H. vaginalis*, only one developed clinical signs and symptoms of BV. In contrast, 11 out of 15 volunteers who were inoculated vaginally with the vaginal secretions of symptomatic donors (screened for other genital tract infections) developed disease [34]. A PubMed search using the term Faecal Microbiome Transplantation demonstrated that in 2012, there were only 22 publications rising to a peak of 1348 in 2024 and 1093 so far in 2025, with a total of 7969 from 2012 to the present. In contrast, a PubMed search using the term vaginal microbiome transplantation demonstrated that between 2003 and 2018, there were only 15 publications on VMT rising to a peak of 25 in 2024 and 13 so far in 2025, with a total of 125 from 2003 to the present. We have a long way to go compared to FMT research, but we feel that VMT will provide a rich vein of research to mine for future investigators.

Research into the vaginal microbiome has increased exponentially in recent years, largely due to technical innovations and a decrease in analytical costs. The results of this research have provided details of microbial communities, their interactions, and the effects on their hosts and other microbes. However, molecular studies of bacteriophages have concentrated on understanding mechanisms by which bacteriophages exploit the intracellular environment of their hosts, but these are binary interactions of how bacteriophage infect bacteria and bacteria infect eukaryotic host cells. Recent studies have demonstrated how tripartite interactions between bacteriophages, bacteria, and the eukaryotic host affect the dynamics and outcome of each component. What becomes evident is that bacteriophages modulate bacterial infections along a spectrum from positive to negative impacts on the mammalian host [143]. Pregnant women and their unborn children are particularly vulnerable to bacterial infection. The physiological changes in pregnancy affect the way women respond to such infections and the treatment options available to clinicians. Antibiotics are still considered the best option for active infections and prophylaxis for the prevention of potential infections, such as recto-vaginal colonisation by *Streptococcus agalactiae* colonisation prior to birth, and the risk of early-onset group B streptococcal disease [38]. The effect of such antibiotic use on the developing fetus, however, is still largely unknown and may cast a long shadow throughout childhood and beyond due to atopy, asthma, allergy, and obesity [144]. An ideal antibacterial therapy for administration during pregnancy would be one that is highly specific for its target, leaving the surrounding microbiota intact. This raises and explores the potential for use of bacteriophage therapy as an alternative to antibiotics during the antenatal period. However, information on the potential for the use of phage therapeutics in pregnancy is lacking [145]. Accordingly, further research is necessary to understand the interaction between bacterial, fungal, phage, and viral microbiomes, together with host genetics, immune status, and environmental factors if we are to provide personalised medical diagnostics and interventions that will improve women’s health [69].

## Figures and Tables

**Table 1:** Bacteria and other microorganisms detected and used to diagnose BV and other causes of vaginal dysbiosis (adapted from Lamont et al. [25]).

	NuSwab **	SureSwab	BD MAX Vaginal Panel ***	MDL BV Panel	AmpliSens *	Hologic Aptima BV
**Positive** **Predictors of BV**						
*Atopobium vaginae* #	+	+	+	+	+	+
*Gardnerella vaginalis*	−	+	+	+	+ *	+
*Megasphaera* spp. (types 1 or 2)	+	+	+	+	−	−
BVAB-2 ##	+	−	+	−	−	−
**Negative** **Predictors of BV**						
*Lactobacillus acidophilus*	−	+	_	−	*	−
*Lactobacillus crispatus*	+	+	+	−	*	+
*Lactobacillus jensenii*	−	+	+	−	*	+
*Lactobacillus gasseri*	−	−	−	−	−	+

BV, bacterial vaginosis; BVAB, bacterial vaginosis-associated bacterium; * The AmpliSens assay was set up to detect the genus *Lactobacillus* rather than specific species of *Lactobacilli*. *G. vaginalis* clades 1 and 2 and *A. vaginae* were detected, and total bacteria for the diagnosis of BV was used. ** An extended version also detects *Candida albicans*, *Candida glabrata*, *Chlamydia trachomatis*, *Neisseria gonorrhoea*, and *Trichomonas vaginalis*. *** Also detects (i) *T. vaginalis*, (ii) Candida Group (*C. albicans*, *Candida tropicalis*, *Candida parapsilosis*, and *Candida dubliniensis*), (iii) *C. glabrata*, and (iv) *Candida krusei*. # Now known as *Fannyhessea vaginae* [42]. ## Now known as *Oscillospiraceae bacterium strain CHIC02* [43]. +: plus, −: minus.

**Table 2:** Numbers and percentages per population of PTB regionally and globally. Adapted from March of Dimes Foundation’s White Paper on preterm birth [71].

Region	Number of PTBs Annually	PTB Rate (%)
Asia	6,907,000	9.1
Africa	4,047,000	11.9
Latin America/Caribbean	933,000	8.1
North America	480,000	10.6
Europe	466,000	6.2
Oceania	20,000	6.4
World Total	12,870,000	9.6

**Table 3:** (**a**) Mortality by gestational age of liveborn infants [adapted from Fellman et al. [73]]. (**b**) Neonatal morbidity by gestational age among survivors [adapted from Fellman et al. [73]].

**(a)**						
**Outcome**	**22 w**	**23 w**	**24 w**	**25 w**	**26 w**	**Total**
Delivery Room Death	55%	16%	6.9%	2%	0%	8.2%
Early NND (<7 d)	80%	39%	21%	11%	9.2%	22%
NND (<28 d)	88%	46%	29%	14%	13%	26%
Infant Death (0–365 d)	90%	48%	33%	19%	15%	30%
**(b)**						
**Morbidity**	**22 w**	**23 w**	**24 w**	**25 w**	**26 w**	**Total**
IVH (>grade 2)	20%	19%	10%	12%	5.2%	10%
ROP (>grade 2)	80%	62%	48%	32%	19%	34%
Severe BPD	40%	26%	31%	29%	17%	25%
PVL	0%	9.4%	6.2%	5.4%	4.5%	5.6%
NEC	0%	1.9%	9.4%	6.0%	5.1%	5.8%
No Major Morbidity @ 365 days	2%	8.9%	21%	37%	54%	32%

w = completed weeks of gestation; d = days; NND = neonatal death; IVH = intraventricular haemorrhage; ROP = retinopathy of prematurity; BPD = Bronchopulmonary Dysplasia; PVL = Periventricular Leucomalacia; NEC = Necrotising Enterocolitis.

**Table 4:** The aetiology of preterm birth (adapted from Lettieri et al.) [76].

Factors	%
Faulty placentation	**50**
Intrauterine infection	**38**
Immunological factors	**30**
Cervical incompetence	**16**
Uterine factors	**14**
Maternal factors	**10**
Trauma and surgery	**8**
Foetal anomalies	**6**

## Data Availability

No new data were created or analyzed in this study. Data sharing is not applicable to this article.

## Abbreviations

AMR	Antimicrobial resistance
BV	Bacterial vaginosis
BVAB	Bacterial vaginosis-associated bacteria
CDC	Centres for disease control
CE-IVD	Conformité Européenne—in vitro diagnostic
CI	Confidence intervals
CST	Community state types
EBV	Epstein–Barr virus
FDA	Food and Drug Administration
FMT	Faecal microbiome transplantation
GAIN(Act)	Generating Antibiotic Innovation Now
GBS	Group B streptococcus
H2O2	Hydrogen peroxide
HIV	Human immunodeficiency virus
HMP	Human microbiome project
HPV	Human papilloma virus
HR-HPV	High-risk human papilloma virus
HSV	Herpes simplex virus
IOM	Institute of Medicine
IUCD	Intrauterine contraceptive device
LM	Late miscarriage
MGD	Millenium development goals
MoD	March of Dimes
NSV	Non-specific vaginitis
OR	Odds ratio
PPROM	Preterm prelabour rupture of the membranes
PTB	Preterm birth
SPTL	Spontaneous preterm labour
Preterm	Labour
STI	Sexually transmitted infections
VMT	Vaginal microbiome transplantation
WSW	Women who have sex with women

## References

[1] Gasanov U., Hughes D., Hansbro P.M. (2005). Methods for the isolation and identification of *Listeria* spp. and *Listeria monocytogenes*: A review. FEMS Microbiol. Rev..

[2] Summers W.C. (2001). Bacteriophage therapy. Annu. Rev. Microbiol..

[3] Górski A., Miedzybrodzki R., Borysowski J., Weber-Dabrowska B., Lobocka M., Fortuna W., Letkiewicz S., Zimecki M., Filby G. (2009). Bacteriophage therapy for the treatment of infections. Curr. Opin. Investig. Drugs.

[4] Principi N., Silvestri E., Esposito S. (2019). Advantages and Limitations of Bacteriophages for the Treatment of Bacterial Infections. Front. Pharmacol..

[5] Rodríguez C., Romero E., Garrido-Sanchez L., Alcaín-Martínez G., Andrade R.J., Taminiau B., Daube G., García-Fuentes E. (2020). Microbiota Insights in Clostridium Difficile Infection and Inflammatory Bowel Disease. Gut Microbes.

[6] Liu K., Wang C., Zhou X., Guo X., Yang Y., Liu W., Zhao R., Song H. (2024). Bacteriophage therapy for drug-resistant Staphylococcus aureus infections. Front. Cell. Infect. Microbiol..

[7] Walsh C. (2003). Where will new antibiotics come from?. Nat. Rev. Microbiol..

[8] Brives C., Pourraz J. (2020). Phage therapy as a potential solution in the fight against AMR: Obstacles and possible futures. Humanit. Soc. Sci. Commun..

[9] Pirnay J.P. (2020). Phage Therapy in the Year 2035. Front. Microbiol..

[10] Swidsinski A., Guschin A., Corsini L., Loening-Baucke V., Tisakova L.P., Swidsinski S., Sobel J.D., Dörffel Y. (2022). Antimicrobial Susceptibility of Microbiota in Bacterial Vaginosis Using Fluorescence In Situ Hybridization. Pathogens.

[11] Swidsinski A., Amann R., Guschin A., Swidsinski S., Loening-Baucke V., Mendling W., Sobel J.D., Lamont R.F., Vaneechoutte M., Baptista P.V. (2024). Polymicrobial consortia in the pathogenesis of biofilm vaginosis visualized by FISH. Historic review outlining the basic principles of the polymicrobial infection theory. Microbes Infect..

[12] Ali A., Jørgensen J.S., Lamont R.F. (2022). The contribution of bacteriophages to the aetiology and treatment of the bacterial vaginosis syndrome. Fac. Rev..

[13] Turnbaugh P.J., Ley R.E., Hamady M., Fraser-Liggett C.M., Knight R., Gordon J.I. (2007). The human microbiome project. Nature.

[14] Saraf V.S., Sheikh S.A., Ahmad A., Gillevet P.M., Bokhari H., Javed S. (2021). Vaginal microbiome: Normalcy vs dysbiosis. Arch. Microbiol..

[15] Chee W.J.Y., Chew S.Y., Than L.T.L. (2020). Vaginal microbiota and the potential of *Lactobacillus* derivatives in maintaining vaginal health. Microb. Cell Fact..

[16] Pendharkar S., Skafte-Holm A., Simsek G., Haahr T. (2023). Lactobacilli and Their Probiotic Effects in the Vagina of Reproductive Age Women. Microorganisms.

[17] Heczko P.B., Giemza M., Ponikiewska W., Strus M. (2024). Importance of Lactobacilli for Human Health. Microorganisms.

[18] Redondo-Lopez V., Cook R.L., Sobel J.D. (1990). Emerging role of lactobacilli in the control and maintenance of the vaginal bacterial microflora. Rev. Infect. Dis..

[19] Lamont R.F., Sobel J.D., Akins R.A., Hassan S.S., Chaiworapongsa T., Kusanovic J.P., Romero R. (2011). The vaginal microbiome: New information about genital tract flora using molecular based techniques. BJOG.

[20] Ravel J., Gajer P., Abdo Z., Schneider G.M., Koenig S.S., McCulle S.L., Karlebach S., Gorle R., Russell J., Tacket C.O. (2010). Vaginal microbiome of reproductive-age women. Proc. Natl. Acad. Sci. USA.

[21] Nouioui I., Carro L., García-López M., Meier-Kolthoff J.P., Woyke T., Kyrpides N.C., Pukall R., Klenk H.P., Goodfellow M., Göker M. (2018). Genome-Based Taxonomic Classification of the Phylum Actinobacteria. Front. Microbiol..

[22] Lyon L.M., Marroquin S.M., Thorstenson J.C., Joyce L.R., Fuentes E.J., Doran K.S., Horswill A.R. (2025). Genome-wide mutagenesis identifies factors involved in MRSA vaginal colonization. Cell Rep..

[23] Hu B.F., Hua C.Z., Sun L.Y., Chao F., Zhou M.M. (2021). Microbiological Findings of Symptomatic Vulvovaginitis in Chinese Prepubertal Girls. J. Pediatr. Adolesc. Gynecol..

[24] Chaudhuri M., Chatterjee B.D., Banerjee M. (1998). A clinicobacteriological study on leucorrhoea. J. Indian Med. Assoc..

[25] Lamont R.F., van den Munckhof E.H., Luef B.M., Vinter C.A., Jørgensen J.S. (2020). Recent advances in cultivation-independent molecular-based techniques for the characterization of vaginal eubiosis and dysbiosis. Fac. Rev..

[26] Cocomazzi G., De Stefani S., Del Pup L., Palini S., Buccheri M., Primiterra M., Sciannamè N., Faioli R., Maglione A., Baldini G.M. (2023). The Impact of the Female Genital Microbiota on the Outcome of Assisted Reproduction Treatments. Microorganisms.

[27] Ntuli L., Mtshali A., Mzobe G., Liebenberg L.J., Ngcapu S. (2022). Role of Immunity and Vaginal Microbiome in Clearance and Persistence of Human Papillomavirus Infection. Front. Cell. Infect. Microbiol..

[28] Schwebke J.R. (2003). Gynecologic consequences of bacterial vaginosis. Obstet. Gynecol. Clin. N. Am..

[29] Cohen C.R., Duerr A., Pruithithada N., Rugpao S., Hillier S., Garcia P., Nelson K. (1995). Bacterial vaginosis and HIV seroprevalence among female commercial sex workers in Chiang Mai, Thailand. AIDS.

[30] Cohen C.R., Lingappa J.R., Baeten J.M., Ngayo M.O., Spiegel C.A., Hong T., Donnell D., Celum C., Kapiga S., Delany S. (2012). Bacterial vaginosis associated with increased risk of female-to-male HIV-1 transmission: A prospective cohort analysis among African couples. PLoS Med..

[31] Lamont R.F., Critchley H., Bennett P., Thornton S. (2004). Bacterial Vaginosis. Preterm Birth.

[32] Piddock L.J. (2012). The crisis of no new antibiotics—What is the way forward?. Lancet Infect. Dis..

[33] Workowski K.A., Bachmann L.H., Chan P.A., Johnston C.M., Muzny C.A., Park I., Reno H., Zenilman J.M., Bolan G.A. (2021). Sexually Transmitted Infections Treatment Guidelines, 2021. MMWR Recomm. Rep..

[34] Gardner H.L., Dukes C.D. (1955). Haemophilus vaginalis vaginitis: A newly defined specific infection previously classified non-specific vaginitis. Am. J. Obstet. Gynecol..

[35] Vodstrcil L.A., Muzny C.A., Plummer E.L., Sobel J.D., Bradshaw C.S. (2021). Bacterial vaginosis: Drivers of recurrence and challenges and opportunities in partner treatment. BMC Med..

[36] Amabebe E., Anumba D.O.C. (2020). Female Gut and Genital Tract Microbiota-Induced Crosstalk and Differential Effects of Short-Chain Fatty Acids on Immune Sequelae. Front. Immunol..

[37] Meyn L.A., Krohn M.A., Hillier S.L. (2009). Rectal colonization by group B Streptococcus as a predictor of vaginal colonization. Am. J. Obstet. Gynecol..

[38] Kenchington A.L., Lamont R.F. (2017). Group B streptococcal immunisation of pregnant women for the prevention of early and late onset Group B streptococcal infection of the neonate as well as adult disease. Expert Rev. Vaccines.

[39] Macklaim J.M., Clemente J.C., Knight R., Gloor G.B., Reid G. (2015). Changes in vaginal microbiota following antimicrobial and probiotic therapy. Microb. Ecol. Health Dis..

[40] Antonio M.A., Hawes S.E., Hillier S.L. (1999). The identification of vaginal *Lactobacillus* species and the demographic and microbiologic characteristics of women colonized by these species. J. Infect. Dis..

[41] Tachedjian G., Aldunate M., Bradshaw C.S., Cone R.A. (2017). The role of lactic acid production by probiotic *Lactobacillus* species in vaginal health. Res. Microbiol..

[42] Brochu H.N., Zhang Q., Song K., Wang L., Deare E.A., Williams J.D., Icenhour C.R., Iyer L.K. (2025). Characterization of vaginal microbiomes in clinician-collected bacterial vaginosis diagnosed samples. Microbiol. Spectr..

[43] Osei Sekyere J., Oyenihi A.B., Trama J., Adelson M.E. (2023). Species-Specific Analysis of Bacterial Vaginosis-Associated Bacteria. Microbiol. Spectr..

[44] Deng T., Song X., Liao Q., Zheng Y., Sun H., Zhang L., Chen X. (2025). Best among the key molecular diagnostic markers of bacterial vaginosis. AMB Express.

[45] Marrazzo J.M., Koutsky L.A., Eschenbach D.A., Agnew K., Stine K., Hillier S.L. (2002). Characterization of vaginal flora and bacterial vaginosis in women who have sex with women. J. Infect. Dis..

[46] Muzny C.A., Sobel J.D. (2025). Bacterial Vaginosis—Time to Treat Male Partners. N. Engl. J. Med..

[47] Vodstrcil L.A., Plummer E.L., Fairley C.K., Hocking J.S., Law M.G., Petoumenos K., Bateson D., Murray G.L., Donovan B., Chow E.P.F. (2025). Male-Partner Treatment to Prevent Recurrence of Bacterial Vaginosis. N. Engl. J. Med..

[48] Di Pietro M., Filardo S., Sessa R. (2025). Cervicovaginal microbiota in Chlamydia trachomatis and other preventable sexually transmitted infections of public health importance: A systematic umbrella review. New Microbiol..

[49] Alisoltani A., Manhanzva M.T., Potgieter M., Balle C., Bell L., Ross E., Iranzadeh A., du Plessis M., Radzey N., McDonald Z. (2020). Microbial function and genital inflammation in young South African women at high risk of HIV infection. Microbiome.

[50] Ng S.C., Hart A.L., Kamm M.A., Stagg A.J., Knight S.C. (2009). Mechanisms of action of probiotics: Recent advances. Inflamm. Bowel Dis..

[51] Villion M., Moineau S. (2009). Bacteriophages of *Lactobacillus*. Front. Biosci..

[52] Van de Wijgert J., Verwijs M.C., Agaba S.K., Bronowski C., Mwambarangwe L., Uwineza M., Lievens E., Nivoliez A., Ravel J., Darby A.C. (2020). Intermittent Lactobacilli-containing Vaginal Probiotic or Metronidazole Use to Prevent Bacterial Vaginosis Recurrence: A Pilot Study Incorporating Microscopy and Sequencing. Sci. Rep..

[53] Hilton E., Isenberg H.D., Alperstein P., France K., Borenstein M.T. (1992). Ingestion of yogurt containing *Lactobacillus acidophilus* as prophylaxis for candidal vaginitis. Ann. Intern. Med..

[54] Kiliç A.O., Pavlova S.I., Ma W.G., Tao L. (1996). Analysis of Lactobacillus phages and bacteriocins in American dairy products and characterization of a phage isolated from yogurt. Appl. Environ. Microbiol..

[55] Pavlova S.I., Kiliç A.O., Mou S.M., Tao L. (1997). Phage infection in vaginal lactobacilli: An in vitro study. Infect. Dis. Obstet. Gynecol..

[56] Jakobsen R.R., Haahr T., Humaidan P., Jensen J.S., Kot W.P., Castro-Mejia J.L., Deng L., Leser T.D., Nielsen D.S. (2020). Characterization of the Vaginal DNA Virome in Health and Dysbiosis. Viruses.

[57] Macklaim J.M., Gloor G.B., Anukam K.C., Cribby S., Reid G. (2011). At the crossroads of vaginal health and disease, the genome sequence of *Lactobacillus iners* AB-1. Proc. Natl. Acad. Sci. USA.

[58] Verwijs M.C., Agaba S.K., Darby A.C., van de Wijgert J. (2020). Impact of oral metronidazole treatment on the vaginal microbiota and correlates of treatment failure. Am. J. Obstet. Gynecol..

[59] Blackwell A.L. (1999). Vaginal bacterial phaginosis?. Sex. Transm. Infect..

[60] Kilic A.O., Pavlova S.I., Alpay S., Kilic S.S., Tao L. (2001). Comparative study of vaginal *Lactobacillus* phages isolated from women in the United States and Turkey: Prevalence, morphology, host range, and DNA homology. Clin. Diagn. Lab. Immunol..

[61] Marrazzo J.M., Antonio M., Agnew K., Hillier S.L. (2009). Distribution of genital *Lactobacillus* strains shared by female sex partners. J. Infect. Dis..

[62] Pavlova S.I., Tao L. (2000). Induction of vaginal *Lactobacillus* phages by the cigarette smoke chemical benzo[a]pyrene diol epoxide. Mutat. Res..

[63] European Medicines Agency (2016). Guideline on the Use of Phthalates as Excipients in Human Medicinal Products.

[64] Kurilovich E., Geva-Zatorsky N. (2025). Effects of bacteriophages on gut microbiome functionality. Gut Microbes.

[65] Garneau J.E., Moineau S. (2011). Bacteriophages of lactic acid bacteria and their impact on milk fermentations. Microb. Cell Fact..

[66] Federici S., Nobs S.P., Elinav E. (2021). Phages and their potential to modulate the microbiome and immunity. Cell. Mol. Immunol..

[67] Wilde J., Slack E., Foster K.R. (2024). Host control of the microbiome: Mechanisms, evolution, and disease. Science.

[68] Zhu J., Ananthaswamy N., Jain S., Batra H., Tang W.C., Lewry D.A., Richards M.L., David S.A., Kilgore P.B., Sha J. (2021). A Universal Bacteriophage T4 Nanoparticle Platform to Design Multiplex SARS-CoV-2 Vaccine Candidates by CRISPR Engineering. bioRxiv.

[69] Vieira-Baptista P., De Seta F., Verstraelen H., Ventolini G., Lonnee-Hoffmann R., Lev-Sagie A. (2022). The Vaginal Microbiome: V. Therapeutic Modalities of Vaginal Microbiome Engineering and Research Challenges. J. Low. Genit. Tract Dis..

[70] Lamont R.F. (2019). Spontaneous preterm labour that leads to preterm birth: An update and personal reflection. Placenta.

[71] March of Dimes Foundation March of Dimes White Paper on Preterm Birth: The Global and Regional Toll 2009 [updated 2009/12/16]. http://www.marchofdimes.org/materials/white-paper-on-preterm-birth.pdf.

[72] Costeloe K., Hennessy E., Gibson A.T., Marlow N., Wilkinson A.R. (2000). The EPICure study: Outcomes to discharge from hospital for infants born at the threshold of viability. Pediatrics.

[73] Fellman V., Hellstrom-Westas L., Norman M., Westgren M., Kallen K., Lagercrantz H., Marsal K., Serenius F., Wennergren M. (2009). One-year survival of extremely preterm infants after active perinatal care in Sweden. JAMA.

[74] Petrou S., Abangma G., Johnson S., Wolke D., Marlow N. (2009). Costs and Health Utilities Associated with Extremely Preterm Birth: Evidence from the EPICure Study. Value Health.

[75] Behrman R.E., Butler A.S., Institute of Medicine (US) Committee on Understanding Premature Birth and Assuring Healthy Outcomes (2007). Preterm Birth: Causes, Consequences, and Prevention.

[76] Lettieri L., Vintzileos A.M., Rodis J.F., Albini S.M., Salafia C.M. (1993). Does “idiopathic” preterm labor resulting in preterm birth exist?. Am. J. Obstet. Gynecol..

[77] Seo K., McGregor J.A., French J.I. (1992). Preterm birth is associated with increased risk of maternal and neonatal infection. Obstet. Gynecol..

[78] Hay P.E., Lamont R.F., Taylor-Robinson D., Morgan D.J., Ison C., Pearson J. (1994). Abnormal bacterial colonisation of the genital tract and subsequent preterm delivery and late miscarriage. BMJ (Clin. Res. Ed.).

[79] Hay P.E., Morgan D.J., Ison C.A., Bhide S.A., Romney M., McKenzie P., Pearson J., Lamont R.F., Taylor-Robinson D. (1994). A longitudinal study of bacterial vaginosis during pregnancy. Br. J. Obstet. Gynaecol..

[80] Lamont R.F. (2015). Advances in the Prevention of Infection-Related Preterm Birth. Front. Immunol..

[81] Rosenstein I.J., Morgan D.J., Lamont R.F., Sheehan M., Dore C.J., Hay P.E., Taylor-Robinson D. (2000). Effect of intravaginal clindamycin cream on pregnancy outcome and on abnormal vaginal microbial flora of pregnant women. Infect. Dis. Obstet. Gynecol..

[82] Lamont R.F., Nhan-Chang C.L., Sobel J.D., Workowski K., Conde-Agudelo A., Romero R. (2011). Treatment of abnormal vaginal flora in early pregnancy with clindamycin for the prevention of spontaneous preterm birth: A systematic review and metaanalysis. Am. J. Obstet. Gynecol..

[83] Finnstrom O., Olausson P.O., Sedin G., Serenius F., Svenningsen N., Thiringer K., Tunell R., Wennergren M., Wesstrom G. (1997). The Swedish national prospective study on extremely low birthweight (ELBW) infants. Incidence, mortality, morbidity and survival in relation to level of care. Acta Paediatr..

[84] Kekki M., Kurki T., Pelkonen J., Kurkinen-Raty M., Cacciatore B., Paavonen J. (2001). Vaginal clindamycin in preventing preterm birth and peripartal infections in asymptomatic women with bacterial vaginosis: A randomized, controlled trial. Obstet. Gynecol..

[85] Gosmann C., Anahtar M.N., Handley S.A., Farcasanu M., Abu-Ali G., Bowman B.A., Padavattan N., Desai C., Droit L., Moodley A. (2017). *Lactobacillus*-Deficient Cervicovaginal Bacterial Communities Are Associated with Increased HIV Acquisition in Young South African Women. Immunity.

[86] Da Costa A.C., Moron A.F., Forney L.J., Linhares I.M., Sabino E., Costa S.F., Mendes-Correa M.C., Witkin S.S. (2021). Identification of bacteriophages in the vagina of pregnant women: A descriptive study. BJOG.

[87] Zhang H.T., Wang H., Wu H.S., Zeng J., Yang Y. (2021). Comparison of viromes in vaginal secretion from pregnant women with and without vaginitis. Virol. J..

[88] Happel A.U., Balle C., Maust B.S., Konstantinus I.N., Gill K., Bekker L.G., Froissart R., Passmore J.A., Karaoz U., Varsani A. (2021). Presence and Persistence of Putative Lytic and Temperate Bacteriophages in Vaginal Metagenomes from South African Adolescents. Viruses.

[89] Madere F.S., Sohn M., Winbush A.K., Barr B., Grier A., Palumbo C., Java J., Meiring T., Williamson A.L., Bekker L.G. (2022). Transkingdom Analysis of the Female Reproductive Tract Reveals Bacteriophages form Communities. Viruses.

[90] Kaelin E.A., Skidmore P.T., Łaniewski P., Holland L.A., Chase D.M., Herbst-Kralovetz M.M., Lim E.S. (2022). Cervicovaginal DNA Virome Alterations Are Associated with Genital Inflammation and Microbiota Composition. mSystems.

[91] Wang J., Xiao L., Xiao B., Zhang B., Zuo Z., Ji P., Zheng J., Li X., Zhao F. (2022). Maternal and neonatal viromes indicate the risk of offspring’s gastrointestinal tract exposure to pathogenic viruses of vaginal origin during delivery. mLife.

[92] Li Y., Cao L., Han X., Ma Y., Liu Y., Gao S., Zhang C. (2023). Altered vaginal eukaryotic virome is associated with different cervical disease status. Virol. Sin..

[93] Britto A.M.A., Siqueira J.D., Curty G., Goes L.R., Policarpo C., Meyrelles A.R., Furtado Y., Almeida G., Giannini A.L.M., Machado E.S. (2023). Microbiome analysis of Brazilian women cervix reveals specific bacterial abundance correlation to RIG-like receptor gene expression. Front. Immunol..

[94] Da Costa A.C., Bortoletto P., Spandorfer S.D., Tozetto-Mendoza T.R., Linhares I.M., Mendes-Correa M.C., Witkin S.S. (2023). Association between torquetenovirus in vaginal secretions and infertility: An exploratory metagenomic analysis. Am. J. Reprod. Immunol..

[95] Hugerth L.W., Krog M.C., Vomstein K., Du J., Bashir Z., Kaldhusdal V., Fransson E., Engstrand L., Nielsen H.S., Schuppe-Koistinen I. (2024). Defining Vaginal Community Dynamics: Daily microbiome transitions, the role of menstruation, bacteriophages, and bacterial genes. Microbiome.

[96] Li C., Jin S., Lv O., Wang G., Zhang Y., Li S., Zhang W., Long F., Shen Z., Bai S. (2024). Comparative analysis of the vaginal bacteriome and virome in healthy women living in high-altitude and sea-level areas. Eur. J. Med. Res..

[97] Huang L., Guo R., Li S., Wu X., Zhang Y., Guo S., Lv Y., Xiao Z., Kang J., Meng J. (2024). A multi-kingdom collection of 33,804 reference genomes for the human vaginal microbiome. Nat. Microbiol..

[98] Kaelin E.A., Mitchell C., Soria J., Rosa A., Ticona E., Coombs R.W., Frenkel L.M., Bull M.E., Lim E.S. (2024). Longitudinal cervicovaginal microbiome and virome alterations during ART and discordant shedding in women living with HIV. Res. Sq..

[99] Orton K.L., Monaco C.L. (2025). The Vaginal Virome in Women’s Health and Disease. Microorganisms.

[100] Madere F.S., Monaco C.L. (2022). The female reproductive tract virome: Understanding the dynamic role of viruses in gynecological health and disease. Curr. Opin. Virol..

[101] Waldor M.K. (1998). Bacteriophage biology and bacterial virulence. Trends Microbiol..

[102] Sausset R., Petit M.A., Gaboriau-Routhiau V., De Paepe M. (2020). New insights into intestinal phages. Mucosal Immunol..

[103] León-Félix J., Villicaña C. (2021). The impact of quorum sensing on the modulation of phage-host interactions. J. Bacteriol..

[104] Erez Z., Steinberger-Levy I., Shamir M., Doron S., Stokar-Avihail A., Peleg Y., Melamed S., Leavitt A., Savidor A., Albeck S. (2017). Communication between viruses guides lysis-lysogeny decisions. Nature.

[105] Damelin L.H., Paximadis M., Mavri-Damelin D., Birkhead M., Lewis D.A., Tiemessen C.T. (2011). Identification of predominant culturable vaginal *Lactobacillus* species and associated bacteriophages from women with and without vaginal discharge syndrome in South Africa. J. Med. Microbiol..

[106] Gudnadottir U., Debelius J.W., Du J., Hugerth L.W., Danielsson H., Schuppe-Koistinen I., Fransson E., Brusselaers N. (2022). The vaginal microbiome and the risk of preterm birth: A systematic review and network meta-analysis. Sci. Rep..

[107] Wylie K.M., Wylie T.N., Cahill A.G., Macones G.A., Tuuli M.G., Stout M.J. (2018). The vaginal eukaryotic DNA virome and preterm birth. Am. J. Obstet. Gynecol..

[108] Racicot K., Cardenas I., Wünsche V., Aldo P., Guller S., Means R.E., Romero R., Mor G. (2013). Viral infection of the pregnant cervix predisposes to ascending bacterial infection. J. Immunol..

[109] Cardenas I., Means R.E., Aldo P., Koga K., Lang S.M., Booth C.J., Manzur A., Oyarzun E., Romero R., Mor G. (2010). Viral infection of the placenta leads to fetal inflammation and sensitization to bacterial products predisposing to preterm labor. J. Immunol..

[110] Souza E.B., Pinto A.R., Fongaro G. (2023). Bacteriophages as Potential Clinical Immune Modulators. Microorganisms.

[111] Gogokhia L., Round J.L. (2021). Immune-bacteriophage interactions in inflammatory bowel diseases. Curr. Opin. Virol..

[112] Champagne-Jorgensen K., Luong T., Darby T., Roach D.R. (2023). Immunogenicity of bacteriophages. Trends Microbiol..

[113] Van Belleghem J.D., Dąbrowska K., Vaneechoutte M., Barr J.J., Bollyky P.L. (2018). Interactions between Bacteriophage, Bacteria, and the Mammalian Immune System. Viruses.

[114] Fettweis J.M., Serrano M.G., Brooks J.P., Edwards D.J., Girerd P.H., Parikh H.I., Huang B., Arodz T.J., Edupuganti L., Glascock A.L. (2019). The vaginal microbiome and preterm birth. Nat. Med..

[115] Schroven K., Aertsen A., Lavigne R. (2021). Bacteriophages as drivers of bacterial virulence and their potential for biotechnological exploitation. FEMS Microbiol. Rev..

[116] Eskew A.M., Stout M.J., Bedrick B.S., Riley J.K., Omurtag K.R., Jimenez P.T., Odem R.R., Ratts V.S., Keller S.L., Jungheim E.S. (2020). Association of the eukaryotic vaginal virome with prophylactic antibiotic exposure and reproductive outcomes in a subfertile population undergoing in vitro fertilisation: A prospective exploratory study. BJOG.

[117] Tosado-Rodríguez E., Alvarado-Vélez I., Romaguera J., Godoy-Vitorino F. (2024). Vaginal Microbiota and HPV in Latin America: A Narrative Review. Microorganisms.

[118] Akbari E., Milani A., Seyedinkhorasani M., Bolhassani A. (2023). HPV co-infections with other pathogens in cancer development: A comprehensive review. J. Med. Virol..

[119] Lebeau A., Bruyere D., Roncarati P., Peixoto P., Hervouet E., Cobraiville G., Taminiau B., Masson M., Gallego C., Mazzucchelli G. (2022). HPV infection alters vaginal microbiome through down-regulating host mucosal innate peptides used by Lactobacilli as amino acid sources. Nat. Commun..

[120] Nicolò S., Antonelli A., Tanturli M., Baccani I., Bonaiuto C., Castronovo G., Rossolini G.M., Mattiuz G., Torcia M.G. (2023). Bacterial Species from Vaginal Microbiota Differently Affect the Production of the E6 and E7 Oncoproteins and of p53 and p-Rb Oncosuppressors in HPV16-Infected Cells. Int. J. Mol. Sci..

[121] Fan Y., Gu Y., Xian Y., Li Q., He Y., Chen K., Yu H., Deng H., Xiong L., Cui Z. (2024). Efficacy and safety of different drugs for the treatment of bacterial vaginosis: A systematic review and network meta-analysis. Front. Cell. Infect. Microbiol..

[122] Antonio M.A., Meyn L.A., Murray P.J., Busse B., Hillier S.L. (2009). Vaginal colonization by probiotic *Lactobacillus crispatus* CTV-05 is decreased by sexual activity and endogenous Lactobacilli. J. Infect. Dis..

[123] Ngugi B.M., Hemmerling A., Bukusi E.A., Kikuvi G., Gikunju J., Shiboski S., Fredricks D.N., Cohen C.R. (2011). Effects of bacterial vaginosis-associated bacteria and sexual intercourse on vaginal colonization with the probiotic *Lactobacillus crispatus* CTV-05. Sex. Transm. Dis..

[124] Ma S., Wang W., Su Y., Sun W., Ma L. (2023). Antibiotics therapy combined with probiotics administered intravaginally for the treatment of bacterial vaginosis: A systematic review and meta-analysis. Open Med..

[125] Abavisani M., Sahebi S., Dadgar F., Peikfalak F., Keikha M. (2024). The role of probiotics as adjunct treatment in the prevention and management of gynecological infections: An updated meta-analysis of 35 RCT studies. Taiwan J. Obstet. Gynecol..

[126] Liu Y., Zhao X., Wu F., Chen J., Luo J., Wu C., Chen T. (2024). Effectiveness of vaginal probiotics *Lactobacillus crispatus* chen-01 in women with high-risk HPV infection: A prospective controlled pilot study. Aging.

[127] Chow K., Wooten D., Annepally S., Burke L., Edi R., Morris S.R. (2023). Impact of (recurrent) bacterial vaginosis on quality of life and the need for accessible alternative treatments. BMC Womens Health.

[128] Javed A., Parvaiz F., Manzoor S. (2019). Bacterial vaginosis: An insight into the prevalence, alternative treatments regimen and it’s associated resistance patterns. Microb. Pathog..

[129] Gao M., Manos J., Whiteley G., Zablotska-Manos I. (2024). Antibiofilm Agents for the Treatment and Prevention of Bacterial Vaginosis: A Systematic Narrative Review. J. Infect. Dis..

[130] Castro J., Sousa L.G.V., França Â., Podpera Tisakova L., Corsini L., Cerca N. (2022). Exploiting the Anti-Biofilm Effect of the Engineered Phage Endolysin PM-477 to Disrupt In Vitro Single- and Dual-Species Biofilms of Vaginal Pathogens Associated with Bacterial Vaginosis. Antibiotics.

[131] Sousa L.G.V., Pereira S.A., Cerca N. (2023). Fighting polymicrobial biofilms in bacterial vaginosis. Microb. Biotechnol..

[132] Zhu M., Frank M.W., Radka C.D., Jeanfavre S., Xu J., Tse M.W., Pacheco J.A., Kim J.S., Pierce K., Deik A. (2024). Vaginal *Lactobacillus* fatty acid response mechanisms reveal a metabolite-targeted strategy for bacterial vaginosis treatment. Cell.

[133] Fischetti V.A. (2003). Novel method to control pathogenic bacteria on human mucous membranes. Ann. N. Y. Acad. Sci..

[134] Renard A., Diene S.M., Courtier-Martinez L., Gaillard J.B., Gbaguidi-Haore H., Mereghetti L., Quentin R., Francois P., Van Der Mee-Marquet N. (2021). 12/111phiA Prophage Domestication Is Associated with Autoaggregation and Increased Ability to Produce Biofilm in Streptococcus agalactiae. Microorganisms.

[135] Bocanova L., Psenko M., Barák I., Halgasova N., Drahovska H., Bukovska G. (2022). A novel phage-encoded endolysin EN534-C active against clinical strain Streptococcus agalactiae GBS. J. Biotechnol..

[136] Yockey L.J., Hussain F.A., Bergerat A., Reissis A., Worrall D., Xu J., Gomez I., Bloom S.M., Mafunda N.A., Kelly J. (2022). Screening and characterization of vaginal fluid donations for vaginal microbiota transplantation. Sci. Rep..

[137] Meng Y., Sun J., Zhang G. (2024). Vaginal microbiota transplantation is a truly opulent and promising edge: Fully grasp its potential. Front. Cell. Infect. Microbiol..

[138] Tuniyazi M., Zhang N. (2023). Possible Therapeutic Mechanisms and Future Perspectives of Vaginal Microbiota Transplantation. Microorganisms.

[139] Yadegar A., Bar-Yoseph H., Monaghan T.M., Pakpour S., Severino A., Kuijper E.J., Smits W.K., Terveer E.M., Neupane S., Nabavi-Rad A. (2024). Fecal microbiota transplantation: Current challenges and future landscapes. Clin. Microbiol. Rev..

[140] Leung V., Vincent C., Edens T.J., Miller M., Manges A.R. (2018). Antimicrobial Resistance Gene Acquisition and Depletion Following Fecal Microbiota Transplantation for Recurrent Clostridium difficile Infection. Clin. Infect. Dis. Off. Publ. Infect. Dis. Soc. Am..

[141] DeFilipp Z., Bloom P.P., Torres Soto M., Mansour M.K., Sater M.R.A., Huntley M.H., Turbett S., Chung R.T., Chen Y.B., Hohmann E.L. (2019). Drug-Resistant E. coli Bacteremia Transmitted by Fecal Microbiota Transplant. N. Engl. J. Med..

[142] Happel A.U., Kullin B.R., Gamieldien H., Jaspan H.B., Varsani A., Martin D., Passmore J.S., Froissart R. (2022). In Silico Characterisation of Putative Prophages in Lactobacillaceae Used in Probiotics for Vaginal Health. Microorganisms.

[143] Marchi J., Zborowsky S., Debarbieux L., Weitz J.S. (2023). The dynamic interplay of bacteriophage, bacteria and the mammalian host during phage therapy. iScience.

[144] Milliken S., Allen R.M., Lamont R.F. (2019). The Role of Antimicrobial Treatment During Pregnancy on the Neonatal Gut Microbiome and the Development of Atopy, Asthma, Allergy and Obesity in Childhood. Expert Opin. Drug Saf..

[145] Furfaro L.L., Chang B.J., Payne M.S. (2017). Applications for Bacteriophage Therapy during Pregnancy and the Perinatal Period. Front. Microbiol..

